# c-Met inhibitor NVP-BVU972 induces antiviral protection and suppresses NF-κB-mediated inflammation

**DOI:** 10.3389/fimmu.2025.1651730

**Published:** 2025-08-29

**Authors:** Yunfei Xie, Yang Zhao, Xingyu Chen, Hongli Jia, Xiao Wang, Tianyi Liu, Haocheng Wang, Yang Li, Xuefei Guo, Fuping You

**Affiliations:** ^1^ Institute of Systems Biomedicine, Department of Immunology, School of Basic Medical Sciences, Beijing Key Laboratory of Tumor Systems Biology, NHC Key Laboratory of Medical Immunology, Peking University Health Science Center, Beijing, China; ^2^ State Key Laboratory of Natural and Biomimetic Drugs, School of Pharmaceutical Sciences, Peking University, Beijing, China; ^3^ Department of Microbiology & Infectious Disease Center, School of Basic Medical Sciences, Beijing Key Laboratory of Tumor Systems Biology, NHC Key Laboratory of Medical Immunology, Peking University Health Science Center, Beijing, China

**Keywords:** NVP-BVU972, C-Met inhibitor, broad-spectrum antiviral, NF-κB-mediated inflammation, epigenetic reprogramming

## Abstract

**Introduction:**

Inhibiting viral replication and limiting NF-κB-driven inflammation simultaneously is essential for better antiviral therapy, highlighting the urgent need for a single agent that achieves both functions.

**Methods:**

Here, we reported NVP-BVU972 (NVP), a selective c-Met inhibitor, induced a robust antiviral state and inhibited NF-κB-mediated inflammation.

**Results:**

The dual functions blocked replication of diverse RNA viruses (VSV, EMCV, MHV) and DNA viruses (HSV-1, VACV) and reduced systemic cytokine levels (Il1β, Il6, Tnfα) in vitro and in vivo. Mechanistically, we identified NVP reprogrammed inflammation-related loci by modulating both gene expression and chromatin accessibility, and chaetocin inhibition of H3K9 methylation reversed its antiviral activity.

**Discussion:**

These findings unveil NVP as a promising host-directed agent that simultaneously limits viral propagation and reduces inflammation, and suggest repurposing NVP as a broad-spectrum antiviral.

## Introduction

Viral infections remain a leading cause of significant illness and death worldwide (1–3). Although direct-acting antivirals that target viral polymerases or proteases have improved outcomes in certain infections, they often face limitations such as narrow specificity, rapid resistance development, and little effect on host inflammation (4–6). In severe viral infections such as seasonal influenza, SARS-CoV, and COVID-19, excessive NF-κB-mediated release of proinflammatory cytokines significantly increases tissue damage and mortality (7–11). Nevertheless, immunosuppressive anti-inflammatory therapies such as corticosteroids indeed reduced immune-driven tissue injury but risked weakening antiviral defenses and allowing viral persistence (12, 13). Thus, systemic dexamethasone lowered mortality in severe COVID-19 by calming cytokine storms, yet its broad immunosuppression delayed viral clearance (14–16). Consequently, host-directed strategies that suppress virus replication while restrain excessive inflammation are urgently needed. We summarized currently available antiviral and anti-inflammatory therapies and their limitations in **Supplementary Table S3** (17–21).

Host-directed antivirals (HDAs) target cellular pathways that are essential for viral replication or disease pathogenesis (22–25). HDAs blocked key host processes to stop multiple viruses at once, compared to direct-acting antivirals (DAAs) against viral proteins, HDAs were shown to offer broad-spectrum activity and reduced the risk of resistance (26, 27). Several host pathways were identified as pro-viral or pro-inflammatory during different infection, such as PI3K/Akt, JAK/STAT, and mTOR, and among them the hepatocyte growth factor (HGF) receptor tyrosine kinase c-Met was especially notable (28–30). c-Met was involved in cell survival, proliferation, and motility (31). For instance, studies have shown that certain influenza A virus strains can exploit c-Met signaling to enhance viral entry and replication (32, 33). Activation of c-Met and downstream kinases can also intersect with inflammatory signaling networks, including the NF-κB pathway that drives cytokine production (34). Thus, inhibiting a host kinase like c-Met could, in principle, disrupt viral replication while also attenuating the amplification of injurious inflammation.

NVP-BVU972 (NVP) is a potent and selective small-molecule inhibitor of c-Met originally developed for cancer therapy (35). In mouse and rat oncology studies, NVP showed favorable pharmacokinetic properties and good tolerability, but its pharmacokinetics and safety in humans have not been evaluated. Its potential to simultaneously target viral replication and virus-induced inflammation consequently remained unexplored.

Artificial intelligence (AI) and machine learning have revolutionized drug discovery and repurposing by mining large-scale chemical and biological datasets to predict compound-target interactions and optimize lead candidates (36–39). Our DeepAVC model used deep learning to integrate phenotypic and transcriptomic data for in silico screening of thousands of compounds. With DeepAVC, we ranked NVP among the top candidates, and subsequent *in vitro* validation confirmed its broad-spectrum antiviral efficacy (40). DeepAVC used computational modeling to analyze both compound-target interactions and antiviral phenotypes, enabling a robust screen of thousands of small molecules. This independent analysis provided evidence suggesting that NVP could exhibit broad-spectrum antiviral activity. We consequently hypothesized that NVP could be repurposed as a host-directed therapeutic by simultaneously attenuating NF-κB-driven inflammation and disrupting host pathways essential for viral propagation.

Here, we reported that NVP significantly restricted viral replication across a spectrum of RNA and DNA viruses, while concurrently reducing NF-κB-driven inflammatory responses through epigenetic reprogramming of host immune responses. Utilizing cell culture and murine models, alongside integrated transcriptomic and epigenomic analyses, we demonstrated that NVP induced a robust antiviral state and dampened pathological inflammation by modulating host gene expression and chromatin accessibility at inflammatory loci. These findings identified c-Met inhibition by NVP as a promising therapeutic strategy with substantial translational potential, providing an innovative foundation for repurposing existing anticancer agents to fulfill urgent clinical needs for effective, broad-spectrum antiviral treatments.

## Result

### NVP-BVU972 establishes a host-protective antiviral state

**Figure 1:**
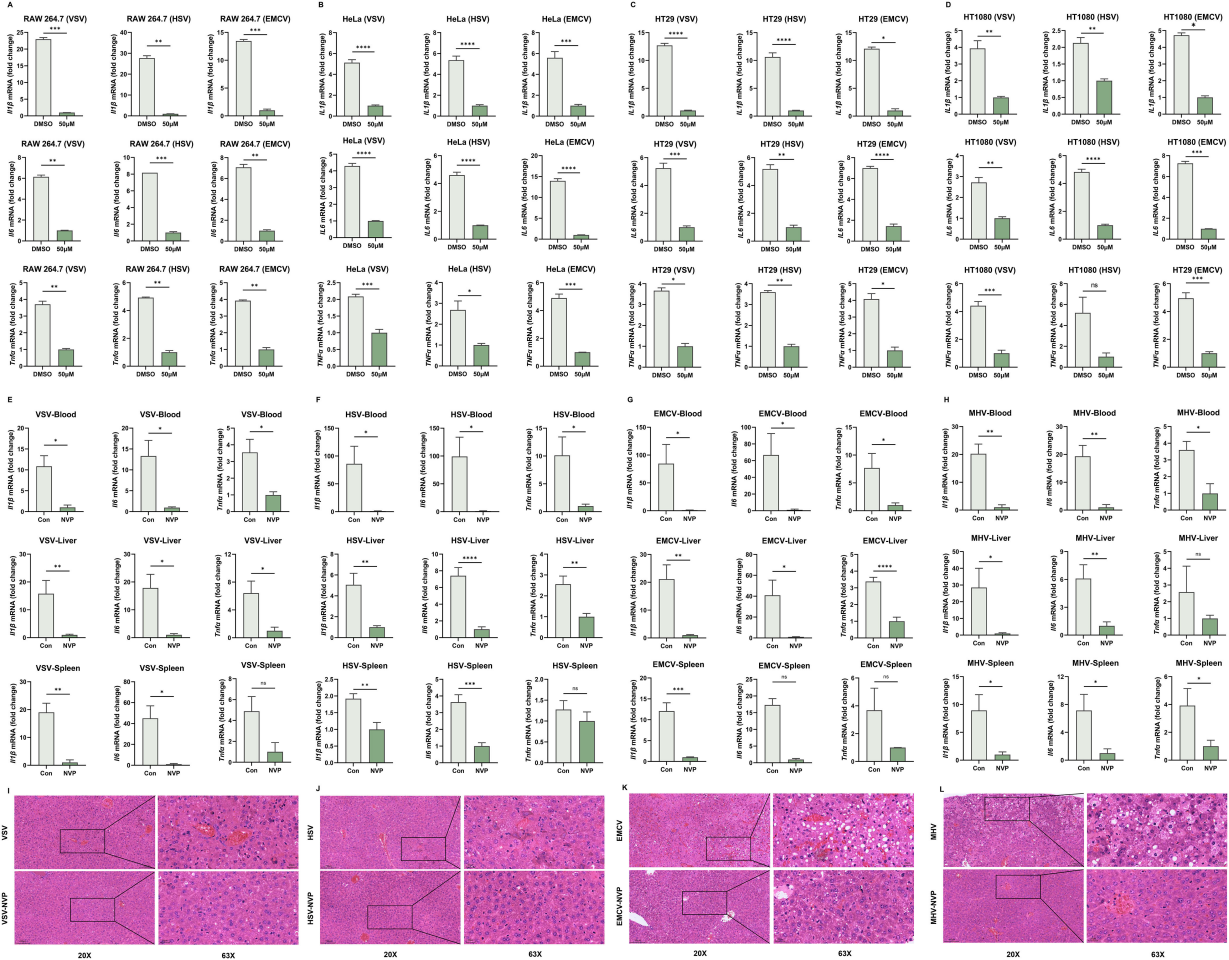
NVP-BVU972 Establishes a Host-Protective Antiviral State. **(A)** RT-qPCR quantification of viral RNA in RAW264.7 treated with DMSO or different concentrations of NVP (25 μM, 50 μM, 100 μM) and infected with MHV, HSV, EMCV and VSV (MOI=0.1). **(B-D)** LPS triggered a Toll-like receptor 4 (TLR4)-mediated signaling cascade that activated the IκB kinase (IKK) complex. IKK then phosphorylated the inhibitory protein IκBα, marking it for degradation and thereby releasing the NF-κB p65 subunit to translocate into the nucleus (a) **(E-F)** Flow cytometric analysis of VSV-GFP infection in HT29, HT1080 and HeLa cells treated with DMSO or 50 µM NVP and infected with VSV-GFP (MOI = 0.1) for 12 hours. Numbers indicated the percentage of GFP positive cells. Data were representative of three independent experiments. **(G)** Representative fluorescence (green) and bright-field images of RAW 264.7 cells treated with DMSO or 50 µM NVP and infected with HSV-GFP, VACV-GFP, VSV-GFP (MOI = 0.1), images were captured at 10× magnification, scale bar = 100 μm. **(H)** Histograms showed flow cytometric quantification of GFP fluorescence (n = 3). **(I)** Dose-dependent inhibition of viral protein expression by NVP. RAW 264.7 cells were treated with the indicated concentrations of NVP and infected with HSV-1, VACV or VSV (MOI = 0.1) for 12 hours, and analyzed by western blot for GFP and Tubulin. **(J)** NVP protected C57BL/6J mice from lethal viral challenge. Kaplan–Meier survival curves of mice infected intraperitoneally with MHV (n = 12), VSV (n = 12), HSV-1 (n = 12) or EMCV (n = 13) and treated daily with PBS or NVP (20 mg/kg). **(K-N)** RT-qPCR quantification of viral RNA in blood and target organs harvested 24 hours post-infection. Y-axis showed fold change of viral gene expression relative to the NVP-treated group, which was set to 1, all values were normalized to GAPDH. Data are shown as mean ± SEM. N.S., not significant, p > 0.05; *p < 0.05; **p < 0.01; ***p < 0.001; ****p < 0.0001.

Based on our previously reported DeepAVC framework, which achieved highly accurate phenotype- and target-guided prediction of broad-spectrum antiviral compounds, we identified NVP-BVU972 (NVP) as a novel candidate and conducted detailed experimental validation of its antiviral efficacy in RAW 264.7 cells. RAW 264.7 cells were concurrently treated for 12 hours with NVP at concentrations of 25, 50, or 100 µM, or DMSO control, and infected at a multiplicity of infection (MOI) of 0.1 with vesicular stomatitis virus (VSV), herpes simplex virus type 1 (HSV-1), mouse hepatitis virus (MHV), or encephalomyocarditis virus (EMCV). RT-qPCR analysis demonstrated a dose-dependent reduction in viral mRNA levels upon NVP treatment compared with DMSO controls, with maximum suppression observed at 100 µM (**Figure 1A**).

To confirm that these antiviral effects extended to human cells, we treated HeLa, HT29, and HT1080 cell lines with 50 μM NVP and concurrently infected with VSV, HSV-1, or EMCV. RT-qPCR quantification indicated a significant 50–70% decrease in viral mRNA levels in NVP-treated cells relative to controls (**Figures 1B-D**). Additionally, flow cytometry analysis at 12 hours post-infection with GFP-expressing VSV (VSV-GFP, MOI=0.1) showed substantially fewer GFP-positive cells in the NVP-treated groups, confirming effective inhibition of viral replication (**Figures 1E, F**).

We further assessed the broad-spectrum antiviral capability of NVP using both DNA viruses (HSV-1 and Vaccinia virus [VACV]) and RNA viruses (VSV) expressing GFP. Fluorescence microscopy (**Figure 1G**) and flow cytometry (**Figure 1H**) consistently revealed significant decreases in GFP expression upon NVP treatment, demonstrating robust suppression of viral replication regardless of virus type.

Dose-dependent antiviral activity was further verified by western blot analysis of GFP protein expression in RAW 264.7 cells infected with HSV, VACV, or VSV (**Figure 1I**). Clear antiviral effects emerged at an NVP concentration of 50 μM, with complete viral suppression achieved at 100 μM.

Encouraged by these compelling *in vitro* findings, we next evaluated the protective effect of NVP *in vivo*. Six-week-old C57BL/6J mice were intraperitoneally infected with MHV, VSV, HSV-1, or EMCV, followed by subsequent treatment with NVP or vehicle control. Survival analysis over a 7-day period revealed significantly improved survival rates in mice receiving NVP treatment compared to untreated controls (**Figure 1J**). Thus, at 24 hours post-infection, RT-qPCR analyses of blood, liver, and spleen tissues from a subset of mice indicated markedly reduced viral levels across all tested viruses in the NVP-treated groups (**Figures 1K-N** and **Supplementary Figure S1A**), further confirming potent antiviral activity *in vivo*.

Together, these results demonstrated that NVP exerted potent, dose-dependent antiviral effects across multiple cell lines and provided broad-spectrum protection against both RNA and DNA viruses *in vivo*.

### NVP-BVU972 suppresses virus-induced inflammatory responses

**Figure 2:**
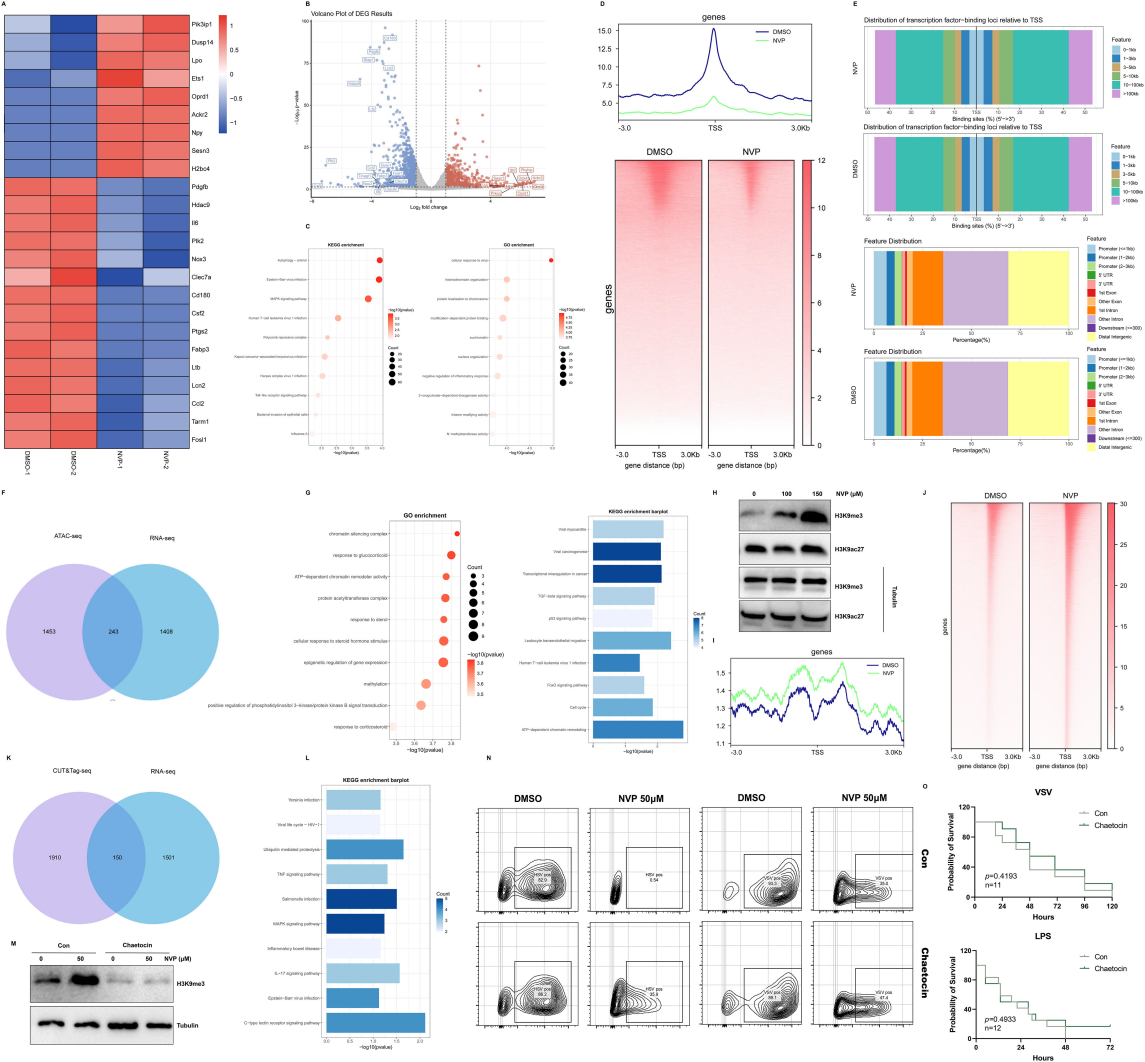
NVP-BVU972 Suppresses Virus-induced Inflammatory Responses. **(A-D)** RT-qPCR quantification of *Il1β*, *Il-6*, *Tnfα* mRNA in RAW264.7, HeLa, HT29, HT1080 cells treated with DMSO or 50 μM NVP and infected with HSV, EMCV and VSV (MOI=0.12). **(E-H)** NVP protected C57BL/6J mice from virus-induced inflammation challenges. Mice infected intraperitoneally with VSV (**E**, n = 12), HSV-1 (**F**, n = 12), EMCV (**G**, n = 13) or MHV (**H**, n = 12) and treated daily with vehicle or NVP (20 mg/kg). RT-qPCR quantification of *Il1β*, *Il-6*, *Tnfα* mRNA in blood and target organs harvested 24 hours post-infection. **(I-L)** Histopathological analysis of tissues from VSV-, HSV-1-, EMCV-, MHV-infected livers of mice treated as in **(E-H)**. Upper panels, low-magnification (20×); lower panels, high-magnification (63×) views of boxed regions. Scale bars, 100 µm (20×) and 20 µm (63×). Data are shown as mean ± SEM. N.S., not significant, p > 0.05.

To determine whether NVP possessed anti-inflammatory activity during viral infections, we first evaluated the expression of pro-inflammatory cytokines at the mRNA level *in vitro*. RAW264.7 macrophages were infected with VSV, HSV-1, EMCV or MHV, and simultaneously treated with 100 µM NVP or DMSO control (**Figure 2A**). Additionally, three human cell lines, including HeLa, HT29 and HT1080 were similarly infected with VSV, HSV-1, or EMCV under identical treatment conditions (**Figures 2B-D**). RT-qPCR analysis at 12 hours post-infection indicated that NVP treatment strongly suppressed virus-induced transcription of pro-inflammatory cytokines *Il6*, *Tnfα*, and *Il1β* compared to the DMSO controls, approaching baseline expression levels in RAW264.7 cells across all viral challenges. Consistent and robust inhibition of these cytokines was also observed across all tested human cell lines, demonstrating that NVP exerted a broad and cell-type-independent anti-inflammatory effect. Additionally, UV−inactivated VSV and poly(I:C) assays showed that NVP directly blocked NF−κB−driven cytokine induction independent of viral replication (**Supplementary Figure S2A, B**).

To further investigate whether NVP-mediated suppression of virus-triggered inflammation could be translated *in vivo*, we infected Six-week-old C57BL/6J mice intraperitoneally with VSV, HSV-1, EMCV, or MHV (**Figures 2E-H**). At 24 hours post-infection, RT-qPCR analysis of pro-inflammatory cytokine mRNA levels in blood, liver, and spleen tissues revealed that NVP administration significantly attenuated the induction of *Il6*, *Tnfα*, and *Il1β* compared to PBS-treated control mice, reflecting systemic suppression of viral inflammation.

Histopathological examination of liver sections using hematoxylin and eosin (H&E) staining further validated the protective effects of NVP *in vivo*. Vehicle-treated, virus-infected mice exhibited pronounced inflammatory cell infiltration, severe hepatocyte damage, and disrupted liver architecture. By contrast, liver tissues from NVP-treated mice displayed significantly reduced inflammatory infiltration, preserved tissue architecture, and minimal hepatocellular injury, consistent with the observed molecular findings (**Figures 2I-L**).

Collectively, these comprehensive *in vitro* and *in vivo* findings established that NVP effectively reduced virus-induced inflammatory responses through suppression of inflammatory cytokine expression, inhibition of NF-κB signaling pathway activation, and alleviation of virus-mediated tissue injury, highlighting its therapeutic potential as a dual antiviral and anti-inflammatory compound.

### NVP-BVU972 reduces LPS-induced inflammation and exhibits low toxicity

Building upon our observations of antiviral and virus-induced anti-inflammatory activity, we next evaluated the direct anti-inflammatory effects of NVP in a classic LPS-induced inflammatory model and assessed subacute safety.

**Figure 3:**
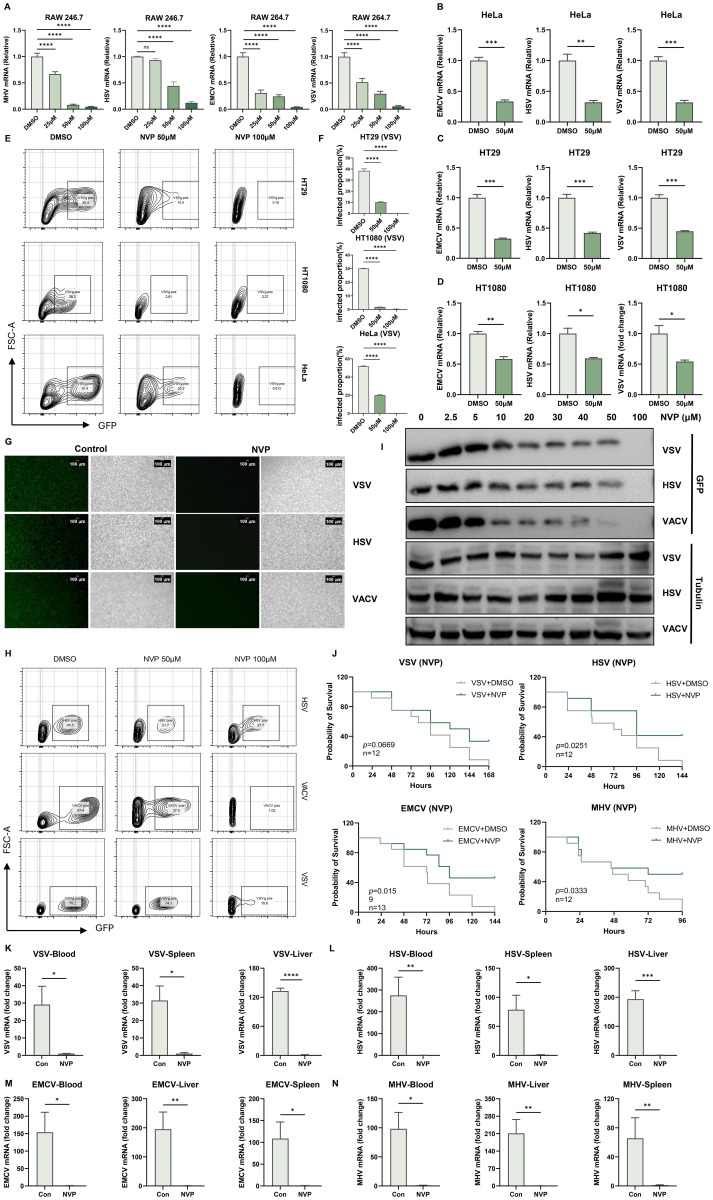
NVP-BVU972 Reduces LPS-Induced Inflammation and Exhibits Low Toxicity. **(A-C)** NVP markedly downregulated the expression of *Il1β*, *Il-6*, *Tnfα*, *Acod1*, *Ccl3*, *Lta*, *Ltb*, *Nos2*, *Ptgs2* mRNA in RAW264.7 cells after 12 hours of LPS stimulation, following treatment with NVP. **(D)** NVP significantly reduced the expression of p-IKKα/β, p-IκBα, t- IκBα, p-P65, t-P65, iNOS, COX2 at the protein level. **(E)** NVP significantly decreased the mortality rate in mice (n=12) following LPS challenge. **(F-K)** RT-qPCR quantification of *Il1β*, *Il-6*, *Tnfα* in blood, heart, spleen, lung, kidney, liver. **(L-M)** Mice received daily intraperitoneal injections of NVP (20 mg/kg) for 14 consecutive days, we compared organ weight **(L)**, organ appearance **(M)** and histopathological structure **(N)** in MOCK, Con (DMSO), NVP. Data are shown as mean ± SEM. N.S., not significant, p > 0.05; *p < 0.05; **p < 0.01; ***p < 0.001; ****p < 0.0001.

First, we treated RAW 264.7 macrophages with 100 µM NVP for 3 hours in the presence of 2 μg/mL lipopolysaccharide from E. coli O55:B5. RT-qPCR analysis showed that LPS significantly induced mRNA expression of multiple inflammatory genes, including *Il1β*, *Il6*, *Tnfα*, *Acod1*, *Ccl3*, *Lta*, *Ltb*, *Nos2*, and *Ptgs2* (**Figures 3A-C**). NVP pretreatment substantially reduced these increases, bringing mRNA levels close to baseline. Importantly, NVP treatment significantly reduced basal *Il6* mRNA levels, whereas *Il1β* remained unchanged and *Tnfα* exhibited only a slight, non−significant decrease (**Supplementary Figure S1C**), demonstrating that NVP did not directly upregulate pro−inflammatory cytokines under resting conditions.

LPS triggered a Toll-like receptor 4 (TLR4)-mediated signaling cascade that activated the IκB kinase (IKK) complex. IKK then phosphorylated the inhibitory protein IκBα, marking it for degradation and thereby releasing the NF-κB p65 subunit to translocate into the nucleus and induce pro-inflammatory gene expression. To explore the underlying mechanism, we performed Western blot analyses of key proteins involved in the NF-κB signaling pathway (**Figure 3D**). LPS stimulation markedly increased phosphorylation of IKKα/β, IκBα, and P65, and significantly induced COX-2 and iNOS protein expression. Treatment with NVP fully blocked these changes without affecting total protein levels of IκBα and P65, indicating that NVP exerted its anti-inflammatory effects by suppressing NF-κB pathway activation.

Next, we examined the anti-inflammatory effects of NVP *in vivo*. Six-week-old C57BL/6J mice received the pretreatment of a single dose of NVP (20 mg/kg, i.p.) followed by a lethal intraperitoneal injection of LPS (20 mg/kg) one hour later. All vehicle-treated mice died within 48 hours, while nearly 50% of the NVP-treated mice survived (**Figure 3E**). To further assess systemic inflammation, we harvested blood, heart, liver, spleen, lung, and kidney tissues 12 hours post-LPS administration and measured inflammatory cytokine mRNA levels. NVP-treated mice showed significantly decreased levels of *Il1β*, *Il6*, and *Tnfα* mRNAs across all tissues compared to controls, demonstrating effective suppression of systemic inflammation (**Figure 3F-K**).

Lastly, we assessed the subacute toxicity of NVP. Mice received daily intraperitoneal injections of NVP (20 mg/kg) for 14 consecutive days. After the treatment period, no significant differences in organ weight (**Figure 3L**), organ appearance (**Figure 3M**), or histopathological structure (heart, liver) (**Figure 3N**) were observed between NVP-treated and control groups. These results confirmed a favorable safety and tolerability profile. In addition, NVP exhibited no significant cytotoxicity in cultured cells as assessed by the CCK-8 assay in RAW264.7, HeLa, HT29, HT1080 cell lines (**Supplementary Figure S1B**).

Collectively, our findings demonstrated that NVP effectively suppressed LPS-induced inflammation by inhibiting NF-κB activation and cytokine production both *in vitro* and *in vivo*, while maintaining excellent safety upon repeated dosing.

### NVP-BVU972 induced transcriptional repression and chromatin remodeling

**Figure 4:**
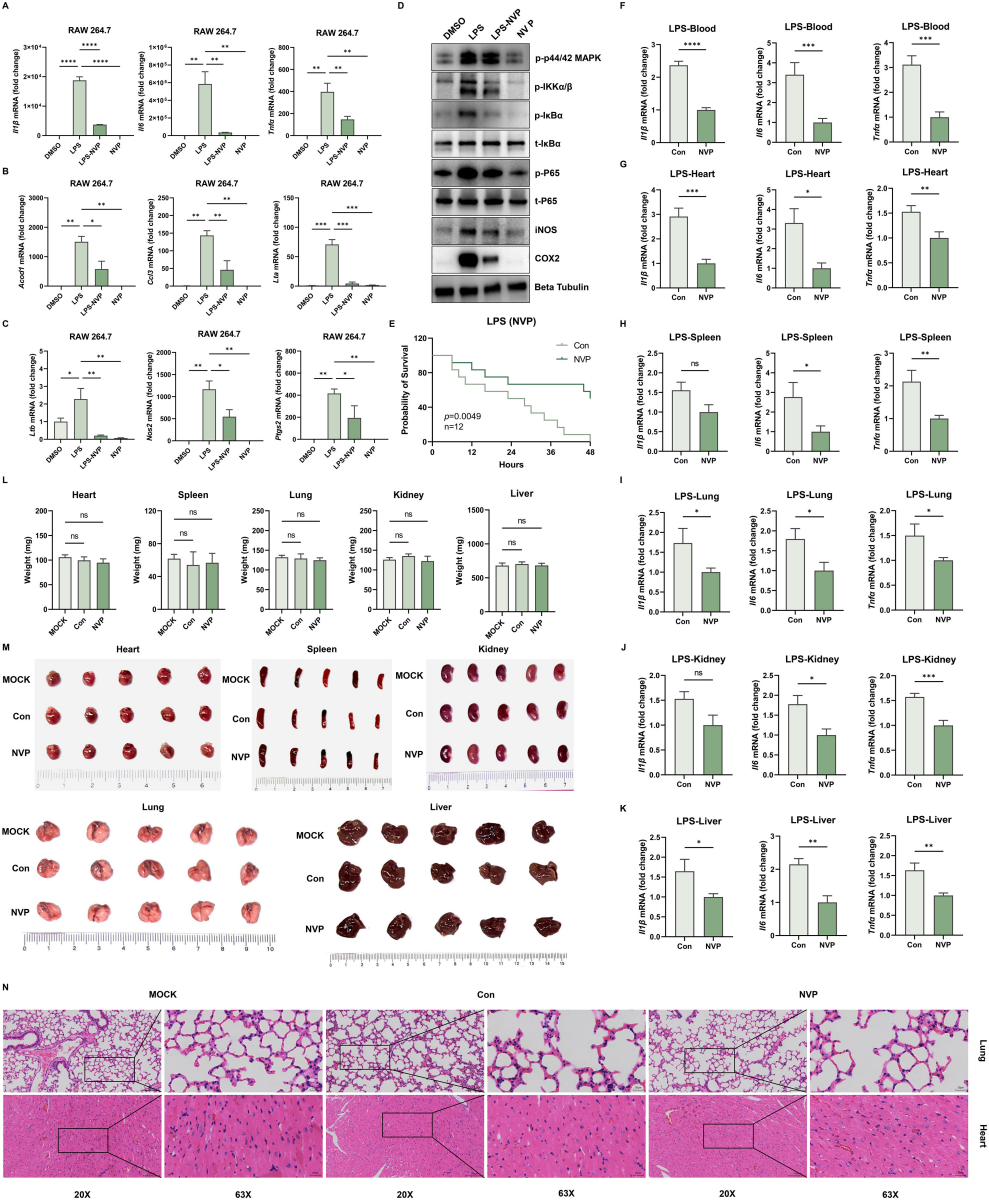
NVP-BVU972 Induced Transcriptional Repression and Chromatin Remodeling. **(A)** Heatmap illustrated the expression of upregulated genes and downregulated genes in the NVP group compared to DMSO groups, specifically those involved in the inflammatory response pathway and anti-viral pathway. **(B)** Volcano plot showed the distribution of differentially expressed genes (DEGs) between the NVP and DMSO groups. **(C)** GO and KEGG enrichment analysis of downregulated DEGs in the NVP group. **(D)** ATAC-seq metaprofile (top) and heatmap (bottom) of chromatin accessibility ±3 kb around peak centers. **(E)** Distance-to-TSS annotation and genomic feature distribution of ATAC peaks in NVP and DMSO samples. **(F)** Venn diagram showed overlap between ATAC-seq and RNA-seq. **(G)** GO and KEGG enrichment analysis of overlap genes in **(F)**. **(H)** RAW264.7 cells treated with NVP and analyzed by western blots for H3K9me3 and H3K9ac27. **(I-J)** Meta-profile **(I)** and heatmap **(J)** of H3K9me3 CUT&Tag signal. **(K)** Venn diagram showed overlap between CUT&Tag-seq and RNA-seq. **(L)** KEGG enrichment analysis of overlap genes in **(K)**. **(M)** Western blot to detect H3K9me3 at protein level treated with NVP (Con) or Chaetocin. **(N)** Flow cytometric analysis of VSV-GFP or HSV-GFP infection in cells treated with Chaetocin or 50 µM NVP for 12 hours. Numbers indicated the percentage of GFP positive cells. Data were representative of three independent experiments. **(O)** C57BL/6J mice pretreated with NVP failed to protect mice from lethal viral challenge and lethal LPS challenge. Data are shown as mean ± SEM. N.S., not significant, p > 0.05; *p < 0.05; **p < 0.01; ***p < 0.001; ****p < 0.0001.

To understand how NVP modulated host responses, RAW264.7 macrophages were treated with 50 µM NVP or DMSO for 12 hours in two independent biological replicates, then we performed bulk RNA sequencing on each sample. Differential expression analysis identified 1402 upregulated and 1706 downregulated genes upon NVP treatment. A heatmap of these differentially expressed genes (DEGs) showed marked suppression of proinflammatory transcripts after NVP treatment (**Figure 4A**). Volcano plot visualization confirmed these cytokine genes among the most significantly downregulated targets (**Figure 4B**). KEGG pathway analysis of all DEGs revealed enrichment in “Autophagy” “Epstein-Barr virus infection” and “MAPK signaling pathway” as the top affected pathways. Gene Ontology enrichment highlighted “cellular response to virus” “heterochromatin organization” “negative regulation of inflammatory response” and “N-methyltransferase activity” (**Figure 4C**). These data indicated that NVP broadly suppressed inflammatory gene programs at the transcriptional level. Notably, the appearance of “histone modifying activity” in GO terms suggested that NVP might alter histone marks to repress inflammatory programs.

To determine whether these transcriptional changes correlated with chromatin accessibility, we carried out ATAC sequencing on cells treated with NVP (50 µM) or DMSO for 12 hours, as in our bulk RNA-seq experiments. Compared to DMSO treatment, NVP induced a net loss of approximately 1696 accessible peaks, with only one peak gained (**Figures 4D, E**). To link these chromatin changes to gene expression, we intersected the list of genes with significantly decreased ATAC peaks and the downregulated transcripts from RNA-seq. This overlap produced 243 genes (**Figure 4F**). GO and KEGG enrichment of this intersected gene set again emphasized “chromatin sliencing complex” “methylation” “viral myocarditis” and “ATP-dependent chromatin remodeling” (**Figure 4G**). Together, these findings suggested that NVP repressed inflammatory gene expression by reducing chromatin accessibility.

Guided by the GO enrichment of histone modifying activity, we next measured global levels of two key histone modifications, H3K9me3 (repressive) and H3K27ac (activating) by Western blot (**Figure 4H**). The result showed that H3K9me3 was increased strongly, whereas H3K27ac levels remained unchanged. This selective enhancement of H3K9me3 supported the hypothesis that NVP promoted deposition of repressive methylation marks at inflammatory gene loci, leading to reduced chromatin accessibility and transcriptional repression. To further confirm that NVP increased H3K9me3 at specific loci, we performed CUT&Tag profiling for this mark in NVP- and DMSO-treated cells for 12 hours, as in our bulk RNA-seq experiments (**Figure 4I, J**). Differential peak calling revealed 2060 loci with elevated H3K9me3 signal under NVP treatment. Intersecting these NVP-induced H3K9me3 peaks with the RNA-seq downregulated genes, 150 genes emerged as candidates whose promoters or enhancers gained repressive methylation concomitant with decreased expression (**Figure 4K**). KEGG analysis of these 150 genes again enriched “Viral life cycle” “MAPK signaling pathway” and “Inflammatory bowel disease” confirming that NVP specifically targeted both antiviral and inflammatory pathways at the epigenetic level (**Figure 4L**).

To test whether H3K9me3 deposition was required for the antiviral activity of NVP, we pretreated cells with Chaetocin, an inhibitor of H3K9 methyltransferases, before NVP exposure and viral infection. Western blots demonstrated that Chaetocin effectively prevented the NVP-induced increase in H3K9me3 (**Figure 4M**). Functionally, flow cytometry showed that VSV-GFP infected cells were significantly higher NVP and Chaetocin co-treated groups compared to NVP alone (**Figure 4N**), indicating that blockade of H3K9 methylation diminished the antiviral efficacy of NVP. Finally, we then assessed the impact of H3K9me3 inhibition on the performance of NVP *in vivo* (**Figure 4O**). In the VSV infection model and LPS-induced inflammation model, survival analysis demonstrated that the addition of Chaetocin abolished the antiviral and anti-inflammation effects of NVP. Addtionally, Chaetocin co-treatment specifically reversed anti-inflammatory actions of NVP, and Chaetocin itself did not non-specifically enhance viral replication or cytokine induction (**Supplementary Figure S2C-F**).

Collectively, NVP induced H3K9me3 deposition at key proinflammatory and pro-viral gene loci, leading to chromatin compaction, reduced accessibility, and transcriptional repression. Inhibition of H3K9 methylation by Chaetocin reversed these epigenetic changes and undermined NVP’ s antiviral and anti-inflammatory efficacy both *in vitro* and *in vivo*, confirming that H3K9me3-mediated chromatin remodeling was essential for the dual therapeutic actions of NVP.

## Discussion

Current antiviral drugs and anti-inflammatory treatments usually work separately, leaving gaps in patient care. Antivirals often targeted specific viruses and can lose effectiveness as viruses mutate (41). At the same time, severe infections like SARS or COVID-19 can trigger harmful cytokine storms, which were treated with steroids that reduced inflammation but also weakened the immune system (42). No host-directed antivirals were currently approved for common infections like influenza, highlighting the need for new approaches. Targeting the hepatocyte growth factor receptor c-Met for antiviral therapy was a novel concept that emerged from our findings. While c-Met inhibitors have traditionally been developed for cancer, very little is known about their effects in viral infections (43). We showed that blocking c-Met both reduced virus replication and suppressed virus-triggered inflammation. Many viruses use c-Met’ s downstream pathways, such as PI3K-Akt, MAPK, or STAT3 to help them grow or keep infected cells alive (30, 33). In some virus-related cancers, like KSHV-driven lymphomas, c-Met supports tumor survival, and blocking it can kill infected cells (44). Overall, NVP can inhibit viral replication and suppress inflammation.

Our work extended that concept to normal viral infection, acute viruses may activate c-Met as part of the body’ s damage response, but this also helped the virus and promoted inflammation. By repurposing the c-Met inhibitor NVP, we shut down both pro-viral and pro-inflammatory signals. Importantly, because c-Met is a host protein, viruses cannot easily develop resistance. Unlike general anti-inflammatory treatments that risk weakening antiviral immunity, c-Met inhibition both reduced harmful inflammation and did not impair viral clearance. This dual benefit made NVP a promising multi-targeted therapy.

We noted that some treatment effects did not reach statistical significance, likely due to dose thresholds or variability. For example, 25 μM NVP failed to significantly reduce HSV viral load in RAW 264.7 cells (**Figure 1A**), suggesting this dose was below the effective threshold for that DNA virus. Similarly, NVP-treated HT1080 cells showed a borderline decrease in HSV-induced *TNF-α* (p=0.052, **Figure 2D**), possibly a Type II error due to small sample size. In LPS-challenged mice, *Il-1β* reductions in spleen (p=0.0665) and kidney (p=0.0877) were also not significant (**Figures 3H, K**), potentially reflecting high baseline variability or organ-specific clearance functions. Among the viruses tested *in vivo*, only MHV elicited a significantly higher splenic *Tnf-α* response, consistent with MHV’s strong spleen tropism, whereas other viruses (VSV, HSV, EMCV) did not.

One of the most important findings was that NVP increased levels of the repressive mark H3K9me3 during viral infection. H3K9me3 was normally used by cells to silence viral DNA and turn off inflammatory genes. For example, the protein KAP1 brought in enzymes that added H3K9me3 to viral genomes, blocking their transcription (45). By boosting H3K9me3, NVP strengthened these natural defenses and shut down viral genes. At the same time, H3K9me3 also turned off host inflammatory genes. In macrophages, for instance, the enzyme Setdb1 added H3K9me3 at the *Il6* promoter, preventing excessive *Il6* production (46). In influenza infection, Setdb2 did the same at the *Cxcl1* gene, reducing neutrophil-driven inflammation (47). In our study, NVP seemed to push the chromatin into a more closed, silenced state at both viral and inflammatory gene sites. Even if NF-κB was active, key inflammatory genes cannot be turned on because their chromatin was decorated with H3K9me3. While NVP initially blocked NF-κB signaling at multiple nodes (**Figure 3D**), the subsequent H3K9me3-mediated repression was focused on specific inflammatory gene promoters, providing sustained, locus-selective dampening of cytokine expression rather than prolonged global pathway shutdown. The idea that an antiviral drug worked partly by changing chromatin marks was new. Because many pathogens and inflammatory signals used the same epigenetic mechanisms, targeting H3K9me3 could explain the broad activity of NVP. It also raised the question of how blocking c-Met lead to H3K9me3 being added to these particular genes, something we need to explore in future work. Based on our current data, we proposed a hypothetical, sequential model in which NVP first acutely blocked NF-κB activation and then promoted H3K9me3−mediated chromatin silencing at the promoters of pro−inflammatory genes, pending further time−course validation.

While our results supported c-Met inhibition as a host-directed antiviral strategy, several limitations need to be mentioned. First, we primarily used cell cultures and acute mouse models, which did not capture the full complexity of human infections. Although we tested multiple RNA and DNA viruses, we did not study chronic or latent infections, such as hepatitis B or latent herpesviruses. It remained unclear whether those viruses responded similarly to c-Met inhibition. Second, repurposing a host kinase inhibitor posed safety challenges. C-Met was involved in normal tissue repair and cell survival, so long-term or widespread inhibition could be toxic (48, 49). In cancer trials, c-Met inhibitors caused side effects such as fatigue, swelling, and organ toxicity, even in very ill patients (43, 50, 51). We used NVP for short-term treatments, its safety with longer courses or in patients with other health issues was unknown. Careful dose optimization and monitoring were essential for any clinical use. Third, our mechanistic insight into H3K9me3 changes was based on correlation. We observed more H3K9me3 marks at certain genes after c-Met inhibition, but we had not yet identified which specific methyltransferase or cofactor was responsible. There may also be other pathways affected by NVP that contributed to its antiviral and anti-inflammatory actions. Fourth, we focused mostly on innate immune responses, such as NF-κB activity in infected cells and macrophages. We did not examine how c-Met inhibition affected adaptive immunity. Since HGF/c-Met signaling can influence macrophage polarization and T-cell functions, blocking c-Met altered these immune cells in ways we have not yet studied (52–54). Finally, while reducing inflammatory cytokines limited tissue damage, lowering inflammation too much slowed viral clearance (55). Some inflammation was needed to recruit immune cells and fought infection. Future studies should look not only at virus levels and cytokines but also at how quickly the body clears the viruses and overall survival in animal models. We also did not examine whether the epigenetic changes we saw, such as increased H3K9me3, persisted after treatment, and if those lasting changes might be helpful or harmful, for example by suppressing needed immune genes. These points reminded us to interpret our findings with caution and highlight areas for further investigation before moving toward clinical trials.

In conclusion, our study introduced c-Met inhibition as a novel host-directed therapeutic strategy that inhibited two central aspects of viral disease, replication and inflammation, through an epigenetic mechanism. While there were clear challenges ahead in translating this approach, the potential benefits exemplified a forward-looking direction in antiviral research. By learning how to modulate host defenses and regulatory networks, we opened the door to treatments that were not only broad-spectrum and resistance-proof but also capable of mitigating the collateral damage of the immune response. This work laid a foundation for future investigations to fully realize the therapeutic promise of targeting c-Met and similar host pathways in the fight against viral infections.

## Materials and methods

### Reagents and antibodies

Rabbit anti-COX2 antibodies were purchased from Wanleibio. Rabbit antibodies against iNOS (catalog no. 80517-1-RR) and Beta Tubulin (catalog no. 10094-1-AP), along with mouse antibodies targeting the DYKDDDDK tag (catalog no. 66008-4-Ig), were procured from Proteintech. Additional antibodies, including rabbit phospho-NF-κB p65 (Ser536) (93H1) (catalog no. 3033), NF-κB p65 (D14E12) XP (catalog no. 8242), phospho-IκBα (Ser32) (14D4) (catalog no. 2859), IκBα (44D4) (catalog no. 4812) and phospho-IKKα (Ser176)/IKKβ (Ser177) (C84E11) (catalog no. 2078), were obtained from Cell Signaling Technology. Secondary detection utilized HRP-conjugated Affinipure Goat Anti-Rabbit IgG (H+L) (catalog no. SA00001-2) and HRP-conjugated Affinipure Goat Anti-Mouse IgG (H+L) (catalog no. SA00001-1), also from Proteintech. Lipopolysaccharides (LPS) (catalog no. HY-D1056) and NVP-BVU972 (catalog no. HY-15456) were sourced from MedChemExpress. Dimethyl sulfoxide (DMSO) (catalog no. D8371) was purchased from Solarbio.

### Cells

The RAW264.7 (RRID: CVCL_0493), HeLa (RRID: CVCL_0030), HT1080 (RRID: CVCL_0316), HT29 (RRID: CVCL_0320), 17-Cl1 (RRID: CVCL_VT75) and Vero (RRID: CVCL_0059) cell lines were sourced from the American Type Culture Collection (ATCC). Cells were authenticated by STR profiling and routinely tested negative for mycoplasma contamination using Mycolor One-Step Mycoplasma Detector (Vazyme, D201-01). RAW264.7, HT1080, 17Cl-1 and Vero were cultured in Dulbecco’s Modified Eagle’s Medium (DMEM) (03.1002C, EallBio), while HeLa and HT29 were maintained in RPMI 1640 (03.4001C, EallBio). All culture media were supplemented with 10% fetal bovine serum (FBS) and 1% penicillin-streptomycin for optimal cell growth and maintenance.

### Virus infection and propagation

Cells at 70-80% confluence were infected with the following viruses at the indicated multiplicity of infection (MOI), vesicular stomatitis virus (VSV, MOI = 0.1), herpes simplex virus 1 (HSV-1, F strain, MOI = 0.5), encephalomyocarditis virus (EMCV, MOI = 0.1), mouse hepatitis virus (MHV, A59 strain, MOI = 0.1) and GFP-expressing viruses, including VSV (MOI=0.1), HSV (MOI=0.1) and vaccinia virus (VACV, MOI=0.1).

Virus stocks were prepared as follows, VSV Indiana strain and VSV-GFP, generously provided by J. Rose (Yale University), were propagated in Vero cells. HSV-1 strain 17, VSV-GFP, HSV-GFP and VACV-GFP were gifts from Zhengfan Jiang (Peking University), and were cultured in the same cell line for viral amplification. MHV-A59 strain (ATCC VR-764) and EMCV (ATCC VR-129B) were obtained from ATCC and propagated in 17Cl-1 and Vero cells, respectively.

### Mice and *in vivo* virus infection

Wild-type (WT) C57BL/6J mice were purchased from the Department of Laboratory Animal Science, Peking University Health Science Center. All animal care and procedures were conducted in accordance with the Guide for the Care and Use of Laboratory Animals by the Chinese Association for Laboratory Animal Science. The protocols were approved by the Animal Care Committee of Peking University Health Science Center (permit number: LA 2016240). Mice were bred and housed under specific pathogen-free conditions at the Laboratory Animal Center of Peking University. Only mice aged 6 to 8 weeks were used in the experiments.

Age- and sex- matched C57BL/6J littermates were used for all *in vivo* experiments. Six-week-old mice were infected with different viruses at a dose of 1×10^8^ plaque-forming units (PFU) per mouse via intraperitoneal injection (IP), and immediately received the first IP dose of NVP (20 mg/kg) or vehicle control, with subsequent treatments administered every other day. Survivals were monitored daily.

We used C57BL/6J mice because they were a well-characterized, immunocompetent inbred strain widely employed in inflammatory and infection models, including LPS-induced sepsis and viral challenge.

### Construction of a sepsis model via LPS injection

This study employed lipopolysaccharide (LPS)-induced sepsis models to investigate systemic inflammatory responses characteristic of sepsis. Male C57BL/6J mice (6–8 weeks old; 20–25 g) were randomized into two groups (n=12 per group): LPS+PBS only and LPS+NVP. A solution of LPS was prepared in sterile saline at a concentration of 20 mg/kg body weight for the WT C57BL/6J mice and administered intraperitoneally using sterile insulin syringes. After LPS injection, a subset of mice received an intraperitoneal injection of NVP. At the same time, the control group was injected with an equivalent volume of PBS. Survival was monitored for 48 hours, blood and lung tissues were subsequently collected at 12 hours post-LPS for downstream analyses.

### Hematoxylin-eosin (H&E) staining

The tissues were quickly placed in cold saline solution and rinsed after they were collected, then fixed in 4% paraformaldehyde, dehydrated, and embedded in paraffin prior to sectioning at 5 mm, and sections were stained with hematoxylin and eosin.

### RNA isolation and RT-qPCR

The total RNA was extracted using TRIzol Reagent (TIANGEN, A0123A01) following the manufacturer’s protocol. Complementary DNA (cDNA) was synthesized from total RNA using HiScript II RT SuperMix (Vazyme, R223-01). mRNA expression levels of viral and immune related genes were analyzed by quantitative reverse transcription PCR (RT-qPCR) using Taq Pro Universal SYBR qPCR Master Mix (Vazyme). The relative expression levels of target genes were normalized to the housekeeping gene Actb, and data were analyzed using the comparative Ct (2^−ΔΔCt) method. The primer sequences used for qPCR analysis in the study can be found in **Supplementary Table S1**.

### Total protein extraction and western blots analysis

The cells were harvested and lysed using ice-cold RAPI buffer (MedChemExpress, HY-K1001), which was supplemented with protease inhibitors for 30 min. Cell lysates were mixed with protein loading buffer and denatured at 100°C for 5 min, followed by centrifugation at 12,000 rpm for 10 min at 4°C. Protein samples were separated by 10% sodium dodecyl sulfate-polyacrylamide gel electrophoresis (SDS-PAGE) and transferred to a nitrocellulose membrane (Beyotime Biotechnology, FFN08) by electroblotting. Membranes were blocked with 5% non-fat milk at room temperature for 1 h and subsequently immunoblotted with indicated primary antibodies (1:1000) at 4°C overnight, followed by incubation with HRP-conjugated secondary antibodies (1:10000) at 37°C for 1 h. Protein signals were detected by using an enhanced ECL system (EallBio, 07.10009-50) according to the manufacturer’s instructions.

### Cell cytotoxicity assay

Target cells were seeded into a 96-well plate at a density of 2×10³ cells per well in 100 μL of complete medium. Cells were treated with increasing concentrations of NVP-BVU972 (MedChemExpress, HY-15456) and incubated at 37°C in a humidified 5% CO^2^ incubator for 48 hours. And then, we added 10 μL of CCK-8 reagent (YEASEN, 40203ES60) to each well and gently shook the plate to mix. After incubation at 37°C for 1 hour, we measured the absorbance at 450 nm using a microplate reader.

### Plaque assay

Confluent monolayers of Vero cells were plated in 24-well plates and infected with 10-fold serial dilutions of virus for 1 hours at 37°C to allow adsorption. The inoculum was then removed, and cells were washed twice with PBS before being overlaid with DMEM containing 0.5% carboxymethyl cellulose (CMC) (Sigma, 419338). Plates were incubated at 37°C for 48 hours to allow plaques to form. The cells were then fixed with 4% paraformaldehyde and stained with 1% crystal violet to visualize plaques. Viral titer was calculated as PFU/mL based on the plaque counts, dilution factor, and inoculum volume.

### Fluorescence assay

Cells were infected with GFP-tagged viruses at MOI of 0.1 for 12 hours. After treatment with NVP, cells were visualized under a Nikon fluorescence microscope equipped with a 488 nm excitation filter and a 510 nm emission filter. Images were captured at a magnification of 100×.

### Flow cytometry analysis

After infection, cells were washed twice with phosphate-buffered saline (PBS) to remove residual virus, followed by trypsinization to generate a single-cell suspension. The cells were collected by centrifugation at 1600 rpm for 5 minutes, and the supernatant was discarded. The resulting cell pellets were resuspended in PBS and transferred to flow cytometry tubes for subsequent analysis. To evaluate the infection efficiency, the percentage of GFP-positive cells was quantified using a flow cytometer (BD Biosciences).

### RNA-seq and data analysis

Total RNA was extracted with the TIANGEN A0123A01 high-throughput kit. Quality control, library preparation, sequencing and downstream analysis were performed by Suzhou GENEWIZ following their standard protocols. Gene counts were generated by featureCounts v2.0.0 and normalized to FPKM. Differential expression was assessed in DESeq2 v1.38.3 using |log_2_FC|>2 and p<0.05.

### ATAC-seq

We performed ATAC-seq using the Hyperactive ATAC-Seq Library Prep Kit (TD711, Vazyme Biotech). Tn5 transposase, preloaded with sequencing adapters, was incubated with isolated nuclei to insert adapters into open chromatin. After PCR amplification with indexed primers, libraries were sequenced by GENEWIZ (Suzhou, China), yielding a genome-wide map of chromatin accessibility.

### CUT&Tag

Cells were captured on ConA-coated magnetic beads and permeabilized with digitonin. After incubation with a primary antibody and a Protein A/G–linked secondary antibody fused to Tn5 transposase, Mg^2+^ activation triggered targeted DNA cleavage and simultaneous adapter insertion. The resulting fragments were PCR-amplified using the Transgen CUT&Tag Library Prep Kit (Vazyme, KP172) and sequenced by GENEWIZ (Suzhou, China).

### Data analysis of ATAC-seq and CUT&Tag

Raw ATAC-seq and CUT&Tag reads (paired-end) from GENEWIZ were first trimmed with Trim-Galore v0.6.4, then aligned to the mouse mm10 genome using Bowtie2 v2.3.5.1 (–very-sensitive -X 2000) and piped through SAMtools v1.10 to produce sorted, indexed BAM files. Peaks were called for each sample with MACS3 v3.0.0a5 (default settings), yielding per-sample BED files (56).

For ATAC-seq, individual peak sets were merged across all samples with Bedtools v2.31.1, and genome-wide coverage tracks were generated in RPKM units using deepTools v3.3.2 (bamCoverage –binSize 100 –normalizeUsing RPKM –effectiveGenomeSize 2864785220 –ignoreForNormalization chrM –extendReads). CUT&Tag signal within called peaks was normalized similarly, and treatment vs. input ratios were computed with bamCompare (–binSize 145 –normalizeUsing RPKM –effectiveGenomeSize 2864785220 –ignoreForNormalization chrM –extendReads –scaleFactorsMethod None) (56).

Differential accessibility/enrichment analyses were performed in R using the csaw package v1.38.0, and peaks were annotated with ChIPseeker v1.34.1. Final track visualization was carried out in IGV v2.17.4.

### Quantification and statistical analysis

Statistical analyses were conducted using Student’s t-test for two-group comparisons (indicated by square brackets) and one-way ANOVA for multi-group experiments. Survival was evaluated by Kaplan–Meier analysis. Data are shown as mean ± SEM (n≥3). N.S.,not significant, *p* > 0.05, *p < 0.05, ** p < 0.01, ***p < 0.001, ****p < 0.0001.

## Data Availability

The datasets presented in this study can be found in online repositories. The names of the repository/repositories and accession number(s) can be found below: https://www.ncbi.nlm.nih.gov/bioproject/PRJNA1272467, PRJNA1272467.

## Supplementary material

Supplementary Figure 1
**(A)** Virus titers in the indicated organs of mice infected with a lethal dose of VSV, HSV-1, EMCV or MHV with or without 24 hours treatment of NVP (as described in the Methods). (B) Cell Viability of RAW 264.7, HT1080, HT29 and HeLa upon NVP treatment. (C) RT-qPCR quantification of *Il1b*, *Il6* and *Tnfa* mRNA in RAW264.7 treated with DMSO or different concentrations of NVP (25 μM, 50 μM, 100 μM). (D) Heatmap illustrated the expression of NF-κB signaling pathway-related genes in the NVP group compared to DMSO group.

Supplementary Figure 2 (A) RT-qPCR quantification of *Il1β*, *Il-6*, *Tnfα* mRNA in RAW264.7 cells treated with DMSO or 50 μM NVP and infected with UV-inactivated VSV (MOI = 0.1). (B) RT-qPCR quantification of *Il1β*, *Il-6*, *Tnfα* mRNA in RAW264.7 cells treated with DMSO or 50 μM NVP and transfected with poly(I:C) (2 µg/mL). (C) RT-qPCR quantification of *Il1β* mRNA and viruses in RAW264.7 cells treated with DMSO, NVP, Chaetocin or NVP+ Chaetocin and infected with HSV and VSV (MOI=0.1). (D) RT-qPCR quantification of *Il1β* mRNA in RAW264.7 cells treated with DMSO, NVP, Chaetocin or NVP+ Chaetocin and treated with LPS. (E) RT-qPCR quantification of *Il1β*, *Il-6*, *Tnfα* mRNA in RAW264.7 cells treated with DMSO or Chaetocin.

Supplementary Table 1 Primers for RT-qPCR.

Supplementary Table 2 Exact p-values of all other comparisons.

Supplementary Table 3 Antiviral and anti-inflammatory therapies.
