# Supplementary figures and images for: c-Met inhibitor NVP-BVU972 induces antiviral protection and suppresses NF-κB-mediated inflammation
Source: Front Immunol. 2025 Aug 29;16:1651730. doi: 10.3389/fimmu.2025.1651730 (PMC12426891; doi:10.3389/fimmu.2025.1651730)

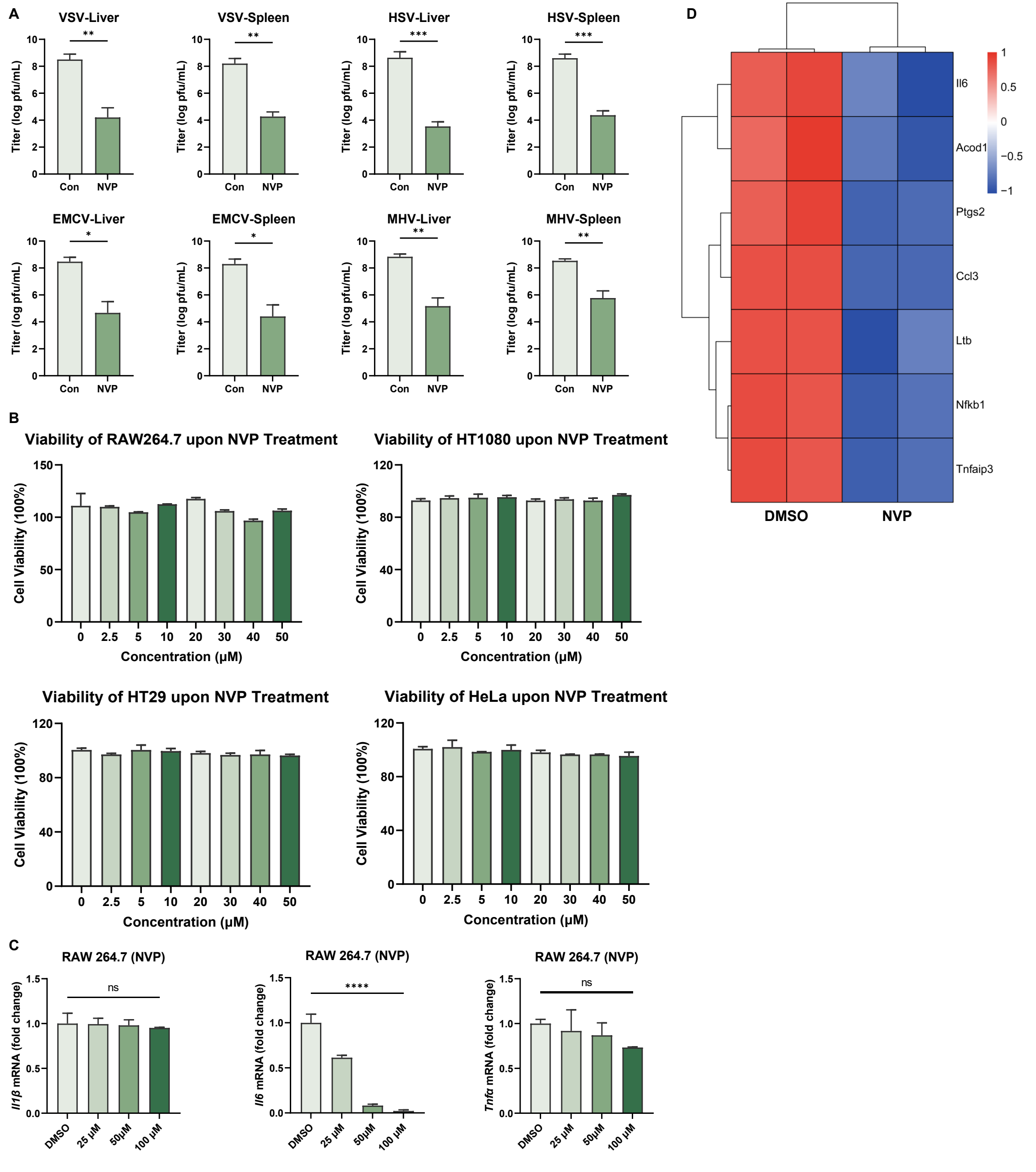

Supplement: Supplementary Figure 1 — (A) Virus titers in the indicated organs of mice infected with a lethal dose of VSV, HSV-1, EMCV or MHV with or without 24 hours treatment of NVP (as described in the Methods). (B) Cell Viability of RAW 264.7, HT1080, HT29 and HeLa upon NVP treatment. (C) RT-qPCR quantification of Il1b, Il6 and Tnfa mRNA in RAW264.7 treated with DMSO or different concentrations of NVP (25 μM, 50 μM, 100 μM). (D) Heatmap illustrated the expression of NF-κB signaling pathway-related genes in the NVP group compared to DMSO group. [file Image1.pdf]

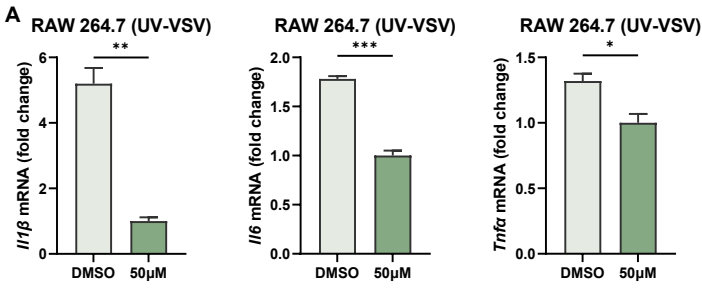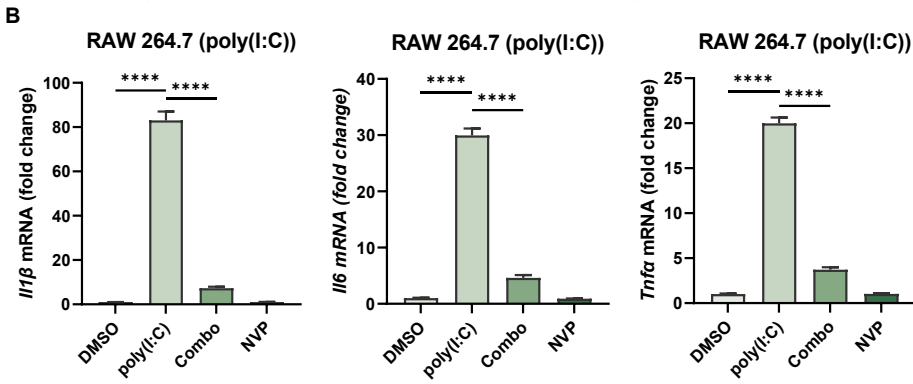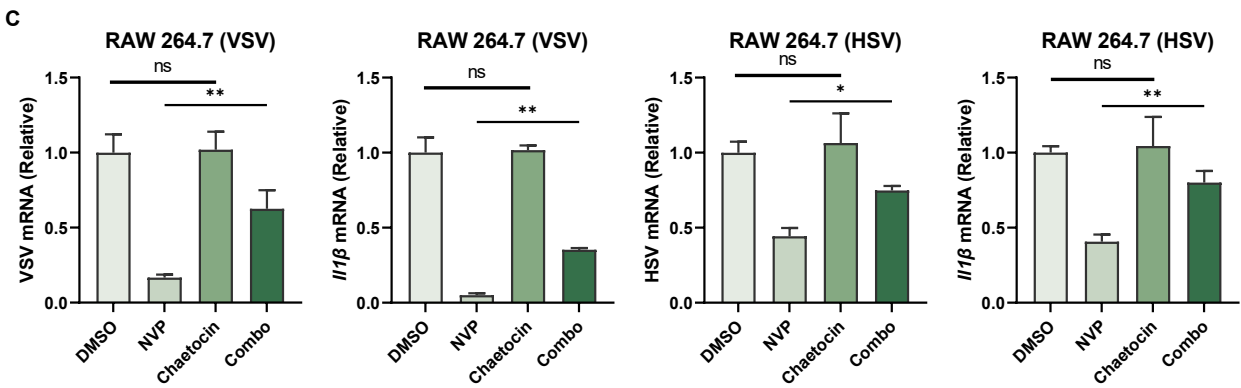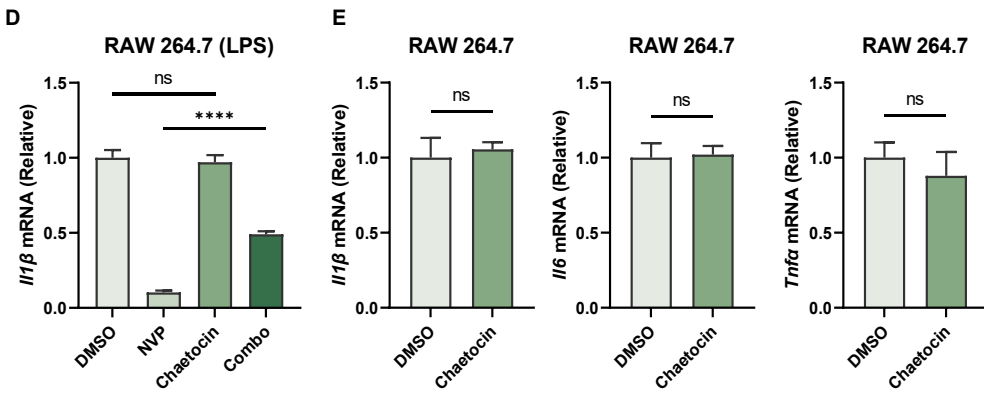

Supplement: Supplementary Figure 2 — (A) RT-qPCR quantification of Il1β, Il-6, Tnfα mRNA in RAW264.7 cells treated with DMSO or 50 μM NVP and infected with UV-inactivated VSV (MOI = 0.1). (B) RT-qPCR quantification of Il1β, Il-6, Tnfα mRNA in RAW264.7 cells treated with DMSO or 50 μM NVP and transfected with poly(I:C) (2 µg/mL). (C) RT-qPCR quantification of Il1β mRNA and viruses in RAW264.7 cells treated with DMSO, NVP, Chaetocin or NVP+ Chaetocin and infected with HSV and VSV (MOI=0.1). (D) RT-qPCR quantification of Il1β mRNA in RAW264.7 cells treated with DMSO, NVP, Chaetocin or NVP+ Chaetocin and treated with LPS. (E) RT-qPCR quantification of Il1β, Il-6, Tnfα mRNA in RAW264.7 cells treated with DMSO or Chaetocin. [file Image2.pdf]
