# Supplementary material for: c-Met inhibitor NVP-BVU972 induces antiviral protection and suppresses NF-κB-mediated inflammation
Source: Front Immunol. 2025 Aug 29;16:1651730. doi: 10.3389/fimmu.2025.1651730 (PMC12426891; doi:10.3389/fimmu.2025.1651730)
Supplement: Supplementary Table 3 — Antiviral and anti-inflammatory therapies. [file Table3.docx]

| Category | Representative drugs | Mechanism | Anti-viral spectrum | Anti-inflammatory | Adverse effect |
| --- | --- | --- | --- | --- | --- |
| Nucleotide and Nucleoside analogues | Remdesivir  Sofosbuvir  Ribavirin | Inhibit viral polymerase; prevent the first step of phosphorylation | Broad, especially RNA viruses | Not directly | Nausea, sleep disturbances, headache, etc. |
| Protease inhibitors(PIs) | Lopinavir  Boceprevir  Nirmatrelvir | Prevent activation by proteolytic cleavage | Specific coronal viruses including HIV/AIDS, HCV and SARS-CoV2 | Some inhibitors can slightly down-regulate IL-6/8 but not main mechanism | Nausea, vomiting and rash, etc. |
| Interferons and immuno-  modulators | Anakinra  Tocilizumab  PEG-IFN | Anti specific proinflammatory cytokines including IL-1α/β, IL-6, etc | Experimentally used on COVID-19, HBV, HCV | Direct downregulation IL-1β, IL-6, TNF-α | Anaphylaxis, angioedema, nasopharyngitis, etc. |
| Viral entry/  fusion inhibitors | Maraviroc  Enfuvirtide  (T20)  Bulevirtide | Inhibit membrane fusion or virus attachment on host cells | Mainly HIV and other coronaviruses | Not directly | Inject site reactions, diarrhea, nausea, fatigue, rash, pyrexia, etc. |
| Neura-  minidase inhibitors | Zanamivir  Oseltamivir  Peramivir | Inhibit the release of newly formed viruses by Preventing the cleavage of hemagglutinin receptors | Influenza | Not directly | Nausea, vomiting, psychiatric effect and renal events, etc. |
